# Using Deliberative Pedagogy as a Tool for Critical Thinking and Career Preparation Among Undergraduate Public Health Students

**DOI:** 10.3389/fpubh.2019.00037

**Published:** 2019-03-04

**Authors:** Denise C. Nelson-Hurwitz, Opal Vanessa Buchthal

**Affiliations:** Office of Public Health Studies, University of Hawai‘i at Mānoa, Honolulu, HI, United States

**Keywords:** public health education, bachelors of public health, undergraduate public health, undergraduate education, curriculum development, high-impact educational practices, career preparation, critical thinking

## Abstract

Engagement of undergraduate public health students in active learning pedagogy has been identified as critical for recruitment, retention, and career preparation efforts. One such tool for engagement that has proven successful in STEM programs is deliberative pedagogy, where it is used to stimulate student interest in research and policy applications of technical course content. Broadly applied, deliberative pedagogy is a consensus model of decision-making, applied as an in-class exercise, where students work in small groups and model a community task force with assigned group roles. In these groups, students collect evidence from literature and media sources, and prepare a consensus response to an assigned question. Here we present an adaptation of this pedagogy to provide undergraduates with the tools needed to actively engage in public health policy and planning work groups. This adaptation is first applied during an introductory public health course, where it is used as a tool for engagement and excitement, and as a critical thinking exercise. It additionally serves as an opportunity for students to apply information literacy skills and engage with research and policy initiatives discussed in class. The same tool is reintroduced prior to graduation in a capstone course, where the emphasis shifts to application of research skills and analytical concepts. The activity is also an opportunity for students to apply professional skills needed for engagement in program development, program evaluation, institutional policy, and legislative advocacy. Through application of this pedagogical tool at two critical time points in an undergraduate curriculum, students develop skills necessary for early career professionals and are better prepared to actively engage in policy and planning as it relates to critical public health initiatives, both locally and globally.

## Background and Rationale

Education of undergraduate students involves a need to introduce, reinforce, and apply basic professional skills, in addition to imparting core content knowledge. Undergraduate education traditionally includes foundational general education courses for this purpose; it is also important for the student's specific degree program to facilitate application of professional skills within the framework of major-specific graduation criteria.

In the last decade there has been a dramatic expansion of public health education from it's historic focus on graduate education and curriculum design into undergraduate programs^1^,^2^ As a result, public health programs and faculty have needed to gain a better understanding of undergraduate student pedagogical needs beyond mastery of introductory public health-specific skills and content knowledge.

Employer assessments of college graduates further supports the need for graduates to demonstrate professional skills in critical thinking, team work and collaboration, and in the ability to apply quality information and data to solve real-world challenges. In a job outlook report conducted and published by the National Association of Colleges and Employers, 82.9% of surveyed respondents reported a need for problem-solving, teamwork, and leadership, with an equivalent 82.9% reporting a demand for graduates to demonstrate an ability to work as a team (1). In a similar study compiled at the request of the Association of American Colleges and Universities (AAC&U), employers reported asking for increased college-level emphasis on integrative learning (73% of respondents), teamwork skills (76%), critical thinking and analytical reasoning skills (73%), and the ability to locate, organize, and evaluate information from multiple sources (70%) (2).

The literature further supports the need to incorporate active learning pedagogy for increased student engagement (3, 4) and performance (5), which may have further implications for student recruitment and retention efforts.

Deliberative pedagogy is a consensus model of decision-making intended to model the function of a community task force (6). Applied in an educational setting as an in-class exercise, students work in small groups in the capacity of assigned group roles. In these groups, students work collaboratively to collect evidence from literature and media sources, then critically appraise and apply this evidence to develop a consensus response to an assigned question. Through employment of this pedagogical practice at two strategic time points in an undergraduate public health curriculum, students develop, and practice workplace-oriented skills in critical thinking, information appraisal and literacy, and teamwork/collaboration. Students are additionally prepared to actively engage in real-world public health challenges and apply quality information to professional engagement in program planning and policy development.

## Pedagogical Framework

Three high-impact educational practices are predominantly reflected in application of deliberative pedagogy. By nature, deliberative pedagogy is a collaborative project, conducted in small student groups, and requires undergraduate students to engage in research practices to find and evaluate credible evidence relevant to their assigned prompt questions. Application of both collaborative projects and undergraduate research are high impact educational practices (7). Focus topics of assigned group prompt questions further reflect values of diversity and global learning, another high-impact educational practice (7). Prompts assigned often focus on public health issues of global importance or implication, or local issues with broad implications. Diversity of opinion is promoted during discussion, most notably represented by the student designated as the “devil's advocate” of the group, as is the practice of applying high quality evidence to support the group's final consensus statement.

Deliberative democracy pedagogy applies several critical component elements (CCEs) identified by the Association of Schools and Programs of Public Health (ASPPH)^3^, and additionally utilizes Liberal Education and America's Promise (LEAP) learning objectives (8, 9). Relevant objectives relate to inquiry and analysis, critical thinking, information literacy, teamwork, problem solving, and social responsivity.

Team-based experiential learning approaches, as applied through deliberative pedagogy, have been shown to be effective in higher education broadly (10, 11), and specifically in application within public health education (12). Peer group learning, applied in small, semi-independent group settings, has been effective in promoting higher order skills in higher education (13), and inquiry-based activities applied in small groups have promoted the mastery of complex material among students (14). Undergraduate programs have been successful in application of a deliberative pedagogy to increase student ability to evaluate and synthesize information, and to actively engage in civic issues engagement (6, 15). The literature further provides support of the effectiveness of deliberative pedagogy in comparison to standard lecture format as evidenced by changes in student self-reported understanding of course content and increases in assessment-based content knowledge (15).

## Learning Environment

The University of Hawai‘i at Mānoa (UHM) is a public research university with enrollment of 17,612 students, and undergraduate students comprising about 73% of enrollees^4^ Public health education began at UHM in 1962 with the offering of graduate-level public health degrees. The Bachelor of Arts in public health (BAPH) degree was added in January 2014, has produced more than 140 graduates, and currently supports approximately 170 declared majors.

Deliberative pedagogy was introduced into public health courses at UHM in fall 2015, and is currently applied at two strategic time points throughout the BAPH curriculum at UHM. It is first introduced during our PH 201 (Introduction to Public Health) course. In this course, the target audience centers on new and prospective undergraduates. As a large lecture format general education course, many, but not all, enrolled students are interested in further pursuing public health as a future field of study. Enrollment in the courses often ranges from 70 to 100 students per class. As an introductory course, the students frequently have no prior exposure to public health concepts and the emphasis of the pedagogy centers on introducing and applying basic public health concepts and skills, including critical thinking skills, collaborative learning, and both collection, and application, of high-quality evidence. The application of this pedagogy further serves to promote awareness among new public health students of current issues of public health concern (e.g., climate change).

Early offerings of the PH 201 course had a small class size, ranging from 30 to 40 students per semester. In-class student debates on public health topics were employed as critical thinking exercises for students when class sizes were small. However, as enrollment in PH 201 increased in subsequent semesters, application of deliberative pedagogy replaced the student debate activities as an approach to scale the activity, while meeting similar critical thinking objectives.

This pedagogy is again applied during our PH 489 (Public Health Undergraduate Capstone Seminar) course. This is often the last public health course students enroll in prior to graduation. As the capstone course, it is intended to integrate students' prior classroom learning with the exposure to public health practice attained through their capstone service-learning or research experience, and to prepare students to enter the workforce or graduate study. As such, the target audience is advanced public health majors who have completed their required public health capstone projects and most, if not all, required coursework in public health. In this course, the emphasis of deliberative pedagogy shifts to center on career preparation and real-world application of public health skills and content. As a university designated writing intensive seminar format course, enrollment is set at a maximum of 20 students per class.

## Learning Objectives

While the deliberative pedagogy and approach is consistently applied throughout the curriculum, it has slightly different goals depending on the course, and subsequently the target audience of students, in which it is being implemented.

Learning objectives of the deliberative pedagogy in PH 201 (Introduction to Public Health) include the following:

1. Introducing and applying critical thinking skills on public health topics
2. Practicing research and investigative skills in population health
3. Identifying and interpreting quality resources in popular and professional literature
4. Exposing students to current controversies and discussions in the field
5. Making connections to public health practice.

Learning objectives of the deliberative pedagogy in PH 489 (Public Health Undergraduate Capstone Seminar) include the following:

1. Reinforcing students' critical thinking skills in public health practice
2. Applying public health skills and concepts to controversies and discussions that students may encounter as early career professionals
3. Gaining practice in teamwork, collaborative problem-solving, and policy development within a public health practice context
4. Integrating public health knowledge with practice in policy analysis
5. Gaining experience in professional communications for policy advocacy

## Pedagogical Format

Deliberative Pedagogy is a consensus decision-making activity modeled on task force protocol. Students work collaboratively in small groups to practice using critical thinking and teamwork to identify information needs and collect evidence, then develop, present, and defend a consensus position on an ethical issue in public health.

Preparation activities require 30–45 min of time allotted in-class to organize the group and plan work. Groups of 4–6 students are pre-assigned into designated group roles by the instructor, primarily for efficiency, but also to challenge students in roles they may not ordinarily have selected for themselves. Each group is assigned a focus question to investigate, and given a worksheet that prompts them to assign team roles, identify the underlying public health issues, develop the group's initial stance on the focus question, identify sub-questions to investigate and potential sources of evidence. Following in-class small group discussion, students are allotted 1–2 weeks for group members to investigate questions identified by the group discussion and collect data.

After the allotted time for research and collection of evidence, groups reconvene in class to review data and develop, present and defend their team's evidence-based consensus statement on the focus question. A final group worksheet documents consensus statement, team member roles, key pieces of evidence with American Psychological Association (APA) citations, and criteria for validity. A full class session is dedicated to this effort and includes 45–60 min for discussion and statement development, as well as 15 min for presentation/large group discussions.

In PH 489, where the deliberative democracy activities also are intended to reinforce teamwork as a critical public health job skill, an additional element is added between the two deliberative democracy activities. After the completion of the first deliberative democracy project, students are guided through an in-class discussion to reflect on the strengths and weaknesses of their teamwork, and uncover for themselves core concepts in team functioning. This understanding is then reinforced through a lecture and guided small-group activities on teamwork. Students are then organized into groups for the second deliberative democracy assignment, and each group is required to develop a team contract that addresses individual responsibilities, team rules and consequences.

Deliberative democracy pedagogy is implemented twice during PH 201- once centered on a topic of environmental health importance, and later centered on a topic of health policy relevance. During PH 489, it is implemented twice- once centered on a topic of legislative advocacy, and later centered on a topic of community health and institutional policy. More specific details describing application of deliberative pedagogy in each PH 201 (Introduction to Public Health) and PH 489 (Public Health Undergraduate Capstone Seminar) are described in Table 1.

**Table 1:** Deliberative democracy implementation overview and sample student group statements.

**Topic**	**Focus questions**	**Sample student group consensus statement**	**Preparation activities**	**Final products**
**PH 201: INTRODUCTION TO PUBLIC HEALTH**				
Climate change and environmental health	*Should the U.S. engage in cap and trade of carbon emissions? Why or why not?*	“The environment wouldn't be in this state at the moment if we were to do what we were supposed to do. If this continues and no change is made, cap and trade of the U.S. is needed to reduce CO_2_ emissions”	Work as a team to: (1) Identify information needs (2) Complete preparation worksheet (3) Gather and review materials from high-quality media and scientific sources	(1) Group presentations of consensus statements (2) Large group discussions (3) Final written worksheet report (4) Short written reflections on assigned group roles
	*What is the most effective action that may be taken by college students to address climate change, both locally and globally?*	“Education is the primary, and most effective action that students can make to address climate change, both locally and globally, starting in the college setting”		
	*Should the U.S. continue to support the Paris Agreement? Why or why not?*	“The U.S. should stay in the [Paris] Agreement because it makes the U.S. look more agreeable to other countries, gives them more credibility as a world leader, and serves as an influencer to lead actions to fight global climate change”		
	*What is the most effective action that may be taken by the U.S. Agriculture industry to address climate change?*	“Planting trees in cities to combat CO_2_ emissions as well as creating a better system of water distribution because there's scarce water resources. We must come up with a better plan to raise livestock. Lastly, we have to focus on soil- it can store 5.5-1.5 billion tons of carbon dioxide globally each year”		
Insurance and health policy	*Should the U.S. expand Medicare to include all U.S. citizens? Why or why not?*	“The U.S. should expand Medicare to include all U.S. citizens with [a] two-tier approach where government taxes [pay] for basic government health care, and citizens can opt to buy a better private insurance. We decided this because compared to other developed nations, we are the last one to not offer universal health care, yet we spend more and have lower life expectancy, and this [Medicare expansion] would improve the overall health of our nation”		
	*Should the State of Hawai‘i revise our health care policy to align with the health care system of Massachusetts? Why or why not?*	“Hawai‘i should adopt Massachusetts's health policy, but potential keep the pre-paid Health Care Act”		
	*How does the health care system work in the State of Hawai‘i and could it work on a national level? Please explain*	“Hawai‘i health care system requires employers to offer affordable health care to their [part-time] employees. This can work on a national-level by following [a] similar model”		
**PH 489: PUBLIC HEALTH UNDERGRADUATE CAPSTONE SEMINAR**				
Legislative Advocacy in Public Health Practice	*Should the Hawai‘i State Legislature pass… (a real bill related to a public health issue that is being debated in the current or upcoming state legislative session. This bill is selected to ensure that it is a topic that has been discussed in public health class, and that it has generated both public controversy and media coverage)*	[Topic: Expansion of SNAP benefit use at farmer's markets] “It's a win-win for the economy, for the affected population, and local farmers—[we are] strong supporters of SB2398, [which] implements a pilot program to allow double bucks programs at local farmers markets and supermarkets”	Work as a team to identify information needs, then gather and review materials from: State legislative record Media sources –State/ national surveillance data –Peer-reviewed literature –Advocacy groups	Group develops a consensus position and formal statement in support or opposition to the legislation Spokesperson delivers and defends the group's oral testimony before a “State Legislator” Completed worksheet with their summary consensus statement, source documentation, and brief reflection on group process
Institutional advocacy in public health practice	*How should our agency use policy advocacy to reduce fall-related injuries among our clients?*	“More education needs to be implemented with care providers—(1) Implementing a fall prevention bill [should] be in effect sooner than 2075; (2) All care providers and health agencies [should] be [at the] forefront to take care of fall prevention”	Work as a team to identify information needs, then gather and review data and policy options from: State/national surveillance data Peer-reviewed literature Media sources Advocacy groups –State or local agencies involved in fall prevention –Individual key informant interviews	Group develops a consensus position/ recommendations on specific fall-prevention policies or initiatives the agency should develop and/or support Spokesperson delivers and defends recommendations before an “Agency Director”

## Outcomes and Assessment Results

Since the deliberative democracy pedagogy is applied in slightly different ways, with variations in learning objectives, during two distinct courses within the UHM public health curriculum, outcome and assessment data are presented here separately.

### PH 201 (Introduction to Public Health) Outcomes and Assessment

When applied in PH 201 (Introduction to Public Health), deliberative democracy pedagogy is a emphasized as a tool through which college freshmen and sophomores with minimal background in public health apply basic public health concepts and skills, including critical thinking skills, collaborative learning, and application of high-quality evidence. The pedagogy also helps to encourage awareness of key public health issues, and promote student enthusiasm in the undergraduate degree program and in public health careers. PH 201 end-of-semester course evaluation data corresponding to the most recent semester, Spring 2018, is provided in Table 2 as compared to aggregated data from the full UHM campus.

**Table 2:** PH 201 end-of-semester course evaluation data—Spring 2018^*^.

	**Agree**	**Strongly agree**	**Combined%**
**I DEVELOPED SKILLS NEEDED BY PROFESSIONALS IN THIS FIELD**			
PH 201	11 (17%)	43 (67%)	84
UHM Aggregate	898 (25%)	2,174 (61%)	86
**I DEVELOPED ENTHUSIASM ABOUT THE COURSE MATERIAL**			
PH 201	15 (24%)	43 (68%)	92
UHM Aggregate	74 (24%)	183 (60%)	84
**MY OPINIONS ABOUT SOME TOPICS CHANGED BECAUSE OF THIS COURSE**			
PH 201	18 (29%)	37 (59%)	88
UHM Aggregate	135 (34%)	187 (47%)	81
**STUDENTS IN THIS COURSE ARE FREE TO DISAGREE AND ASK QUESTIONS**			
PH 201	14 (22%)	48 (74%)	96
UHM Aggregate	1,196 (21%)	3,974 (69%)	90

* *Questions reported were optional additions to end-of-semester course evaluations. UHM Aggregate data reported reflect the UHM respondents among courses where the question was selected and asked of students*.

In coding of open-ended responses on the most current PH 201 course evaluation (Spring 2018, *n* = 64 responses), the top two coded reponses to “what do you feel was the most valuable aspect of this course?” was interactions or discussions (8 responses) and specifically named deliberative democracy discussions (7 responses). In a free comments section, the most common feedback (8 responses) indicated students wanted deliberative democracy activities to be expanded or utilized more frequently in the course.

Anecdotal evidence from end-of-semester interviews conducted with instructors of public health courses taken by students following PH 201 suggests students are able to better distinguish between high and low quality evidence, and are better able to recognize when data are needed to support claims, compared to student demonstration of skills prior to implementation of deliberative democracy pedagogy.

### PH 489 (Undergraduate Public Health Capstone) Outcomes and Assessment

When applied in PH 489 (Undergraduate Public Health Capstone), deliberative democracy pedagogy is a career development tool promoting application of professional skills, and encouraging engagement of early career professionals in policy development and analysis. The pedagogy is intended to strengthen students' awareness of the role of policy advocacy in public health practice, to build students' familiarity with common data sources used in policy analysis, and to reinforce critical-thinking, professional teamwork and professional communication skills related to public health policy advocacy. PH 489 end-of-semester course evaluation data corresponding to the most recent semester, Spring 2018, is provided in Table 3 as compared to aggregated data from the full UHM campus.

**Table 3:** PH 489 end-of-semester course evaluation data—Spring 2018^*^.

	**Agree**	**Strongly agree**	**Combined%**
**I DEVELOPED SKILLS NEEDED BY PROFESSIONALS IN THIS FIELD**			
PH 489	5 (23%)	17 (77%)	100
UHM aggregate	898 (25%)	2,174 (61%)	86
**I DEVELOPED THE ABILITY TO SOLVE REAL PROBLEMS IN THIS FIELD**			
PH 489	7 (32%)	14 (64%)	95
UHM aggregate	512 (25%)	1,261 (61%)	86

* *Questions reported were optional additions to end-of-semester course evaluations. UHM Aggregate data reported reflect the UHM respondents among courses where the question was selected and asked of students*.

Open-ended responses to a question asking students to identify the most valuable parts of the course were coded. More than half (9 out of 14) of the comments identified in-class group activities like the deliberative democracy activities as the most valuable part of the class. They felt that these activities were valuable because they “helped me remember key points about public health,” “involved practice of real-world skills,” or “involved preparation for public health careers.”

Since the BAPH program is relatively new, data gathered from employers of graduates is pending, however, an employer survey is currently being developed with plans for deployment within the academic year.

## Discussion

Deliberative Democracy activities can foster critical thinking and help students make the link between policy and the concrete public health practice experiences (6, 15). Deliberative Democracy activities are closely related to task force or team activities routinely performed in public health practice, and can be an effective tool for career preparation. Learning objectives associated with Deliberative Democracy, also integrate well with public health Bachelor's degree program competencies as outlined by CEPH (Council on Education for Public Health) accreditation criteria (16) D10 (Public Health Bachelor's Degree Foundational Domains), D11 (Public Health Bachelor's Degree Foundational Competencies), and D13 (Public Health Bachelor's Degree Cross-Cutting Concepts and Experiences) as outlined in Tables 4, 5.

**Table 4:** Learning Objectives of Deliberative Pedagogy in PH 201 (Introduction to Public Health) Mapped with CEPH (Council on Education for Public Health) Accreditation Criteria^*^.

**Learning objectives of the deliberative pedagogy in PH 201 (Introduction to Public Health)**	**Relevant public health Bachelor's Degree Foundational Domains (CEPH Criterion D10)**	**Relevant public health Bachelor's Degree Foundational Competencies (CEPH Criterion D11)**	**Relevant public health bachelor's Degree Cross-Cutting Concepts and Experiences (CEPH Criterion D13)**
1. Introducing and applying critical thinking skills on public health topics	The basic concepts, methods and tools of public health data collection, use and analysis and why evidence-based approaches are an essential part of public health practice (2.1, 2.4, 2.5, 2.6)	The ability to locate, use, evaluate, and synthesize public health information	Critical thinking and creativity (3) Ethical decision making as related to self and society (5)
2. Practicing research and investigative skills in population health	The basic concepts, methods and tools of public health data collection, use and analysis and why evidence-based approaches are an essential part of public health practice (2.1, 2.3, 2.4, 2.5)	The ability to locate, use, evaluate, and synthesize public health information	Independent work and a personal work ethic (6) Research methods (10)
3. Identifying and interpreting quality resources in popular and professional literature	The basic concepts, methods and tools of public health data collection, use and analysis and why evidence-based approaches are an essential part of public health practice (2.2, 2.3, 2.4, 2.5)	The ability to locate, use, evaluate, and synthesize public health informationThe ability to communicate public health information, in both oral and written forms, through a variety of media and to diverse audiences	Independent work and a personal work ethic (6) Research methods (10)
4. Exposing students to current controversies and discussions in the field	Basic concepts of legal, ethical, economic and regulatory dimensions of health care and public health policy and the roles, influences and responsibilities of the different agencies and branches of government (8.1, 8.2, 8.4, 8.5)	N/A	Advocacy for protection and promotion of the public's health at all levels of society (1) Ethical decision making as related to self and society (5)
5. Making connections to public health practice	N/A	The ability to communicate public health information, in both oral and written forms, through a variety of media and to diverse audiences	Advocacy for protection and promotion of the public's health at all levels of society (1) Cultural contexts in which public health professionals work (4) Teamwork and leadership (12)

* Numbered values correspond to specific criteria on CEPH Data Templates available https://ceph.org/documents/32/2016templates.xlsx

**Table 5:** Learning objectives of deliberative pedagogy in PH 489 (Undergraduate Public Health Capstone) Mapped with CEPH (Council on Education for Public Health) Accreditation Criteria^*^.

**Learning objectives of the deliberative pedagogy in PH 489 (Undergraduate public health capstone)**	**Relevant public health Bachelor's Degree foundational domains (CEPH Criterion D10)**	**Relevant public health Bachelor's Degree foundational competencies (CEPH Criterion D11)**	**Relevant public health Bachelor's degree cross-cutting concepts and experiences (CEPH Criterion D13)**
1. Reinforcing students' critical thinking skills in public health practice	The basic concepts, methods and tools of public health data collection, use and analysis and why evidence-based approaches are an essential part of public health practice (2.1, 2.4, 2.5, 2.6)	The ability to locate, use, evaluate and synthesize public health information	Critical thinking and creativity (3) Ethical decision making as related to self and society (5)
2. Applying public health skills and concepts to controversies and discussions that students may encounter as early career professionals	The basic concepts, methods and tools of public health data collection, use and analysis and why evidence-based approaches are an essential part of public health practice (2.1, 2.3, 2.4, 2.5)	The ability to locate, use, evaluate and synthesize public health information	Community dynamics (2) Cultural contexts in which public health professionals work (4) Research methods (10)
3. Gaining practice in teamwork, collaborative problem-solving, and policy development within a public health practice context	The basic concepts, methods and tools of public health data collection, use and analysis and why evidence-based approaches are an essential part of public health practice (2.2, 2.3, 2.4, 2.5) Basic concepts of legal, ethical, economic and regulatory dimensions of health care and public health policy and the roles, influences and responsibilities of the different agencies and branches of government (8.1, 8.2, 8.4, 8.5)	The ability to locate, use, evaluate and synthesize public health information The ability to communicate public health information, in both oral and written forms, through a variety of media and to diverse audiences	Community dynamics (2) Cultural contexts in which public health professionals work (4) Professionalism (9) Research methods (10) Systems thinking (11) Teamwork and leadership (12)
4. Integrating public health knowledge with practice in policy analysis	Basic concepts of legal, ethical, economic and regulatory dimensions of health care and public health policy and the roles, influences and responsibilities of the different agencies and branches of government (8.1, 8.2, 8.4, 8.5)	The ability to locate, use, evaluate and synthesize public health information	Advocacy for protection and promotion of the public's health at all levels of society (1) Ethical decision making as related to self and society (5)
5. Gaining experience in professional communications for policy advocacy	Different concepts of public health-specific communication, including technical and professional writing and the use of mass media and electronic technology (9.1, 9.2)	The ability to communicate public health information, in both oral and written forms, through a variety of media and to diverse audiences	Advocacy for protection and promotion of the public's health at all levels of society (1) Cultural contexts in which public health professionals work (4) Professionalism (9) Teamwork and leadership (12)

* Numbered values correspond to specific criteria on CEPH Data Templates available https://ceph.org/documents/32/2016templates.xlsx

Throughout application of deliberative pedagogy at two strategic curriculum time points, student feedback and assessment activities lead to adaptations and evolution of pedagogical application. In working with a large, lecture-format course of introductory public health students within the framework of PH 201, it was clear that careful instruction and repetition was important, as was continuous monitoring during discussions. Preparation for deliberative pedagogy class sessions must include curricula focused on critical thinking, finding quality resources, and evaluating evidence, which were found to be essential in providing an adequate foundation of skills to apply during deliberative pedagogy sessions. The use of multiple focus, or prompt, questions was necessary to expose students to a range of issues related to a central topic, and avoid redundancy during report-back. Logistically, advanced assignment of student groups and designated roles was found to maximize time spent on discussion, rather than logistics. Grading of preparation activities and final products separately was also identified as necessary to address student absences during one of the two designated class sessions.

In applying deliberative pedagogy in a seminar-format course with advanced public health students within the framework of PH 489, we found students required prompting and specific guidance from the instructor to link the in-class deliberative pedagogy policy activities to related content introduced during prior courses. This is further supported by literature articulating the benefits of repetition throughout an undergraduate degree curriculum (17). Logistically, adding a class session between the first and second deliberative pedagogy sessions where students reflect on collaborative team function and discussed professional teamwork skills was found to enhance student skill development in the second session. Specifying a broad range of information sources to consult for policy questions supported advanced students in understanding the range of practical factors agency staff and leadership may weigh in developing an agency response to a public health concern. Finally, deliberative pedagogy sessions were found to have greater salience for advanced students when the connection to public health career skills was made explicit throughout the activity.

Deliberative Democracy pedagogy is successfully applied at UHM during two critical time points in the undergraduate public health curriculum. Data suggests it is successful in meeting intended learning objectives, including the application of critical thinking skills and promoting the linkage between policy and the concrete public health practice experiences. Deliberative Democracy activities are closely related to task force or team activities routinely performed in public health practice, and can be an effective tool for career preparation.

## Data Availability

The datasets generated for this study are available on request to the corresponding author.

## Ethics Statement

This study was carried out in accordance with the recommendations of the University of Hawai‘i (UH) Human Studies Program as exempt from federal regulations pertaining to the protection of human research participants. Authority for the exemption applicable is documented in the Code of Federal Regulations at 45 CFR 46.101(b) 4. The protocol was approved by the Office of Research Compliance, University of Hawai‘i system (Protocol Number 2018-00751).

## Author Contributions

DN-H: initial conception and adaptation of the pedagogy to both courses; DN-H and OB: contributed substantial reformatting of the pedagogy; DN-H: wrote the first draft of the manuscript. Both authors wrote sections of the manuscript, contributed to manuscript revision, read, and approved the submitted version.

### Conflict of Interest Statement

The authors declare that the research was conducted in the absence of any commercial or financial relationships that could be construed as a potential conflict of interest.

## Footnotes

^1^https://bigfuture.collegeboard.org/majors/health-professions-related-clinical-sciences-public-health-public-health

^2^https://www.aspph.org/connect/data-center/

^3^http://www.aspph.org/educate/framing-the-future/

^4^https://manoa.hawaii.edu/about/
